# Levels of Pesticides and Their Metabolites in Wistar Rat Amniotic Fluids and Maternal Urine upon Gestational Exposure

**DOI:** 10.3390/ijerph10062271

**Published:** 2013-06-04

**Authors:** Rossana Bossi, Anne Marie Vinggaard, Camilla Taxvig, Julie Boberg, Eva Cecilie Bonefeld-Jørgensen

**Affiliations:** 1 Department of Environmental Science, Aarhus University, Frederiksborgvej 399, Roskilde 4000, Denmark; 2 National Food Institute, Technical University of Denmark, Department of Toxicology and Risk Assessment, Mørkhøj Bygade 19, Søborg 2860, Denmark; E-Mails: annv@food.dtu.dk (A.M.V.); camta@food.dtu.dk (C.T.); jubo@food.dtu.dk (J.B.); 3 Centre for Arctic Health and Unit for Cellular & Molecular Toxicology, Department of Public Health, Build. 1260, Bartholins Allé 2, Aarhus University, Aarhus C 8000, Denmark; E-Mail: ebj@mil.au.dk

**Keywords:** pesticides, gestational exposure, *in vivo* experiment, Wistar rats

## Abstract

Concentrations of pesticides and selected metabolites in rat urine and amniotic fluid were determined as biomarker upon oral administration of Wistar rats to two pesticide mixtures consisting of three to five pesticides (bitertanol, propiconazole, cypermethrin, malathion, and terbuthylazine). The pesticides and their metabolites were found in rat amniotic fluid and urine, generally in dose-response concentrations in relation to dosage. The measurement of the substances in the amniotic fluid indicated that the fetus was exposed to the pesticides as well as their metabolites. Moreover, the pesticides detected in urine demonstrated the exposure as well as the ability of the rat to excrete these compounds.

## 1. Introduction

The endocrine disrupting effect of a large number of man-made chemicals has been in focus as one of the possible causes of alterations of the reproductive system, such as reproductive organ malformations and low fertility [1]. Several studies *in vitro* and in laboratory animals, as well as investigations carried out on pesticide workers have shown endocrine disrupting effects of several past- and currently-used pesticides [2]. There is growing evidence that gestational exposure to pesticides, even at low concentrations, may lead to damages of the reproductive system. Several studies have e.g., shown a higher incidence of cryptorchidism and hypospadias in sons of women working as gardeners or living in places where pesticides are intensively used [3,4,5]. 

Assessment of exposure to chemicals with potential endocrine disrupting effects during the early phase of the gestation is important since the development of reproductive organs of the fetus occurs in this period [6]. Human *in utero* exposure is commonly estimated on analysis of maternal blood, umbilical cord blood, placental and accompanying fluid concentrations at birth. However, the data obtained at this point are only representative for recent exposure and/or exposure to persistent compounds, which are present in the maternal circulation at equilibrium with the fatty tissues [7,8].

Most of the current used pesticides are non-persistent and are generally excreted within hours or days as water soluble metabolites in the urine. Thus measurements of these compounds in the maternal blood and in cord blood or placental tissue do not necessarily reflect the total fetal exposure during gestation. Measurements of chemicals in meconium and amniotic fluid have been considered indicative biomarkers of direct fetal exposure [9,10]. Sampling of amniotic fluid during the first 20 weeks of gestation will give a better insight of the chemical exposure of the fetus in the most delicate phase of its development. However, sampling of amniotic fluid through amniocentesis during the early stages of the gestation may pose risk to the fetus and thus is not a widely applicable procedure to assess *in utero* exposure to chemicals. 

Organochlorine pesticides (OCs) and other persistent compounds such as polychlorinated biphenyls (PCBs) and polybrominated biphenyl ethers (PBDEs), which are lipophilic and bound to serum lipids, have been detected in amniotic fluid [6,11,12]. Only few data on levels of non-persistent pesticides and metabolites are available from direct analysis of amniotic fluid after amniocentesis [9,13]. 

Among current used pesticides, the exposure to azole fungicides and atrazine has been studied because of their suspected endocrine disrupting effects [14,15,16,17]. Commonly observed effects of azole fungicides were increased gestational length, virilize female pups, and affected steroid hormone levels in fetuses and/or dams. 

The present study is a part of a larger project (HOPE) investigating the potential endocrine disrupting effects of selected currently used pesticides through *in vitro* and *in vivo* experiments. The selection of the test compounds was based on a list of active compounds registered for use in Denmark, the treated area (hectares), and literature data on the compounds endocrine disrupting potential. Based on *in vitro* screening in different cell culture systems for the endocrine disrupting potential of 13 pesticides, five compounds with the strongest endocrine disrupting potential where chosen for mixture analyses *in vitro*, *in vivo* rat and *ex vivo* human placental transfer experiments. The five investigated compounds included the fungicides bitertanol and propiconazole, the insecticides cypermethrin and malathion, and the herbicide terbuthylazine. The selected pesticides were mixed in two different mixtures consisting of three or five pesticides, respectively. The purpose of the present study was to measure the levels of the test pesticides and their metabolites in the amniotic fluids and the maternal urine of orally exposed Wistar rats.

## 2. Materials and Methods

### 2.1. Animals and in utero Exposure

Cypermethrin, PESTANAL^®^, analytical standard (CAS No. 52315-07-8), malathion, PESTANAL^®^, analytical standard (CAS No. 121-75-5), bitertanol PESTANAL^®^, analytical standard (CAS No. 55179-31-2), propiconazole PESTANAL^®^, analytical standard (CAS No. 60207-90-1), and terbuthylazine PESTANAL^®^, analytical standard (CAS No. 5915-41-3) were all purchased from Sigma-Aldrich (St. Louis, MO, USA). The test compounds were dissolved in corn oil (the vehicle, No. C8267-2.5 L) from Sigma-Aldrich.

The animal study was performed under conditions approved by the Danish Animal Experiments Inspectorate, and by the in-house Animal Welfare Committee. The study included 84 time-mated Wistar rats supplied on gestational day (GD) 3 and upon arrival, randomly distributed in pairs and housed under standard conditions: semitransparent polycarbonate cages (15 × 27 × 43 cm) with Aspen bedding situated in an animal room with controlled environmental conditions (12 h light-dark cycles with light starting at 9 p.m., light intensity 500 lux, temperature 21 ± 2 °C, humidity 50% ± 5%, ventilation 8 air changes per hour). A complete ALTROMIN 1314 rodent diet for growing animals and acidified tap water (to prevent microbial growth) was provided *ad libitum*. The animals were observed twice daily for signs of toxicity, and body weights were recorded daily during the dosing period. The day after arrival, *i.e.*, GD 4, animals were weighed and distributed into seven groups of 12 animals, with similar weight distributions. An acclimatization period of 4 days was allowed before starting exposure. Dams were gavaged with the pesticide mixtures daily from gestation day (GD) 7 to 21. The five pesticides were mixed in two different mixtures. A mixture named “mix 3” consisting of: bitertanol, propiconazole, and cypermethrin mixed in the ratio 1:1:1, and a mixture called “mix 5” consisting of all five pesticides mixed in a 1:1:1:1:1 ratio. The maximum dose of each compound in the mixtures, *i.e.*, 10 mg/kg bw/day per compound, was selected on the basis of previous toxicological data, and the highest dose was equivalent to the highest dose at which only subtle effects were expected on maternal body weights and litter size.

Group 1 received vehicle only (corn oil). Group 2, Group 3, and Group 4 received a 1:1:1 mixture of bitertanol, propiconazole and cypermethrin in doses of 1, 3 and 10 mg/kg bw/day per compound. Total doses were 3 mg/kg bw/day, 9 mg/kg bw/day and 30 mg/kg bw/day, respectively. Group 5, Group 6 and Group 7 received a 1:1:1:1:1 mixture of bitertanol, propiconazole, cypermethrin, malathion and terbuthylazine in doses of 1, 3 and 10 mg/kg bw/day per compound, *i.e.*, total doses of 5 mg/kg bw/day, 15 mg/kg bw/day and 50 mg/kg bw/day, respectively. 

At GD 15, six dams from each of the seven groups were placed in metabolic cages immediately after dosing, and urine was collected in cooled containers over a period of 4 h. At the end of the collection period, the samples were stored at −80 °C. At GD 21 dams were weighed, anesthetized in CO_2_/O_2_ and decapitated approximately 2 h after dosing (±30 min). Cesarean sections were performed as previously described in [18]. The uterus was taken out and weighed, and the amniotic fluid from each dam was collected in glass tubes and stored at −80 °C. Rat fetuses each have their own individual placenta and amniotic sac, but amniotic fluid from all fetuses in a litter was pooled.

### 2.2. Analysis of Pesticides and Metabolites

A common analytical method was used for determination of the polar pesticides and their metabolites in the amniotic fluid and the urine. The compounds analyzed included bitertanol, propiconazole, terbuthylazine, desethylterbuthylazine, 2-hydroxyterbuthylazine, malathion dicarboxylic acid (specific metabolite of malathion), and 3-phenoxybenzoic acid (3-PBA, metabolite of cypermethrin) (Table 1). The available volume for the sample was 100 µL for amniotic fluid and 1 mL for urine from each dam. The samples were analyzed with the method from Baker *et al.* [19], slightly modified. The samples were spiked with isotopically labeled standard, and then diluted with 1 mL 0.2 M ammonium acetate buffer solution containing 4,290 activity units of β-glucoronidase and 245 activity units sulfatase. The solution was allowed to incubate at 37 °C overnight. The sample was then extracted by solid phase extraction on C18 cartridges, 500 mg (Discovery, Supelco, Bellefonte, PE, USA). The instrumental analysis was performed with high performance liquid chromatography-tandem mass spectrometry (LC-MS/MS). The LC-MS/MS system consisted of an Agilent 1200 series (Agilent Technologies, St. Clara, CA, USA) and a QTrap 5500 triple quadrupole mass spectrometer (AB Sciex, Framingham, MA, USA) equipped with an electrospray ionization source (ESI) operating in negative or positive mode. Separation of the analytes was achieved on a Hypersil C18 column (250 × 2.1 mm, 5 µm particle size; Phenomenex, Torrance, CA, USA) with 10 µL injection volume. The composition of the mobile phase was 5 mM ammonium acetate (A) and methanol (B) run at a flow rate of 0.2 mL/min with linear gradient. Multiple Reaction Monitoring (MRM) was used to isolate two specific precursor and product ion pairs for each compound. Detection of the analytes was based on retention time and the most abundant mass transition corresponding to an authentic standard. Confirmation of analyte identity was based on the relative response of the secondary mass transition to the primary mass transition. Internal standard method was applied for quantification using isotope labeled standards (propiconazole D_5_, desethylterbuthylazine D_5_ and 3-PBA ^13^C_6_). Calibration standards in the concentration range 1–50 ng/mL and laboratory blanks were analyzed concurrently with unknown samples. The analytical limit of detection (LOD) varied by the analyte and matrix (Table 1). The analysis of cypermethrin required a separate method since this compound is not amenable by LC-MS/MS analysis. Cypermethrin was not expected to be present in the urine samples since this pesticide is rapidly metabolized to non-toxic and more polar metabolites and was therefore analyzed only in amniotic fluid [20,21]. The amniotic fluid samples (800 µL volume) were spiked with ^13^C_6_-cis-permethrin (Cambridge Isotopes Laboratories, Andover, MA, USA) and extracted by liquid-liquid extraction with 2 mL hexane. After centrifugation at 2,800 rpm for 15 min the organic phase was evaporated to dryness and reconstituted in 100 µL isooctane. An Agilent 7890 GC coupled to an Agilent 5975 C mass spectrometer was used for the analysis. The sample was injected in splitless mode and the analytes were separated on a HP-5MS column (30 m, 0.25 mm i.d., 0.25 µm film thickness, Agilent). The GC-MS was operated in selected ion monitoring (SIM) with Negative Chemical Ionization (NCI) using methane as ionization gas. Internal standard method was applied for quantification using ^13^C_6_-cis-permethrin. Calibration standards in the concentration range 25–500 ng/mL and laboratory blanks were analyzed concurrently with unknown samples. The analytical LOD for cypermethrin in amniotic fluid was 0.09 ng/mL (Table 1).

**Table 1:** Recoveries and limits of detection (LOD) for the pesticides and their metabolites in rat urine and amniotic fluid.

Compound	Recovery % (±RSD %) n = 10	Urine LOD (ng/mL)	Amniotic fluid LOD (ng/mL)
Cypermethrin	93.4 (±7.5)	n.a.	0.09
3-PBA (*)	106.5 (±2.2)	0.05	0.60
Bitertanol	96.5 (±9.1)	0.03	0.72
Propiconazole	105.5 (±1.9)	0.03	0.62
Terbuthylazine	103.2 (±4.1)	0.004	0.05
Desethylterbuthylazine (**)	79.9 (±6.2)	0.02	0.21
2-Hydroxyterbuthylazine (**)	91.7 (±6.3)	0.002	0.12
Malathion dicarboxylic acid (***)	19.9 (±10.7)	0.16	0.35

n.a. = not analyzed; (*) metabolite of cypermethrin; (**) metabolite of terbuthylazine; (***) metabolite of malathion.

## 3. Results and Discussion

The amniotic fluid from 69 pregnant Wistar rats and the maternal urine from 32 Wistar rats orally exposed to the pesticide mix-3 (cypermethrin, propiconazole and bitertanol) and the pesticide mix-5 (cypermethrin, propiconazole, bitertanol, terbuthylazine and malathion) were analyzed for the parent pesticides and their metabolites. The metabolites included in the analytical methods have been chosen on the basis of their availability as commercial standards and the possibility to be included in the multi-residues method together with the parent compounds. The pesticide malathion was not included in the analytical method because of interferences from other compounds in the MS-MS analysis. Instead, the metabolite malathion dicarboxylic acid was chosen as marker for malathion exposure since a study performed with rats dosed at three levels with malathion demonstrated that carboxylic acid metabolites account for up to 80% of the total excreted metabolites, with only small amounts of dialkylphosphates [22]. The common metabolite for the triazole fungicides bitertanol and propiconazole, 1,2,3-triazole was initially included in the LC-MS-MS method, but the compound could not be retained on the SPE cartridge and thus excluded from the analytical method. 

The concentrations of the pesticides and their metabolites measured in the rat’s amniotic fluid and maternal urine for each dose of the pesticide mix are given in detailed in Table 2, Table 3, Table 4. The target compounds were not detected in the control group. The concentrations are given as mean of three sets of animals including four animals in each set. All the analyzed parent pesticides and their metabolites were detected in amniotic fluid with average concentrations ranging from 0.1 to 749.1 ng/mL (Table 2, Table 3). Moreover, for all compounds there was a tendency to a higher level upon exposure to the pesticide mix-3 compared to the mix-5. 

Bitertanol was found at relatively higher concentrations in amniotic fluid (range: 19.9–749.1 ng/mL) (Table 2, Table 3) compared to the level in urine (range: 1.3–16.7 ng/mL) (Table 4, Table 5). Studies performed with Wistar rats orally exposed to ^14^C-labelled bitertanol have shown that this compound is rapidly adsorbed after ingestion reaching the maximum concentration in plasma after 3.8 h [23]. Most of the applied radioactivity was excreted in the faeces (90%) and only 7% was excreted with the urine. This is in agreement with the low bitertanol levels measured in urine samples (Table 4, Table 5) in the present study. Biotransformation studies of bitertanol in rats showed that this compound is rapidly metabolized after adsorption from the gastrointestinal tract. However, the high bitertanol levels measured in amniotic fluid in the present study indicate that a significant fraction of the unchanged parent compound is transferred to the maternal circulation and further to the fetus. 

**Table 2:** Mean (±standard deviation) concentrations of the pesticides and the 3-PBA metabolite (ng/mL) in amniotic fluid from Wistar rats orally exposed to mix-3 at three different doses.

Compound	Dose 3 mg/kg (n = 12)	Dose 9 mg/kg (n = 11)	Dose 30 mg/kg (n = 12)
Cypermethrin	4.9 (±4.4)	9.7 (±7.2)	33.3 (±37.6)
3-PBA (*)	31.6 (±14.5)	82.9 (±72.5)	185.3 (±138.0)
Bitertanol	24.5 (12.8)	115.5 (19.9)	749.1 (±420.8)
Propiconazole	0.7 (±0.6)	1.7 (±1.6)	3.3 (±1.8)

(*) metabolite of cypermethrin.

The other triazole fungicide, propiconazole, had the lowest concentration range (0.3–3.3 ng/mL) among the parent pesticides in both amniotic fluid (Table 2,Table 3) and urine (Table 4, Table 5). The low concentrations of propiconazole found in amniotic fluid indicate that the parent pesticide is not transferred into the maternal circulation. A laboratory study with orally exposed rats showed that propiconazole was rapidly adsorbed and metabolized, resulting in a wide array of metabolites isolated from urine [24].

**Table 3:** Mean (±standard deviation) concentrations of the pesticides and metabolites (ng/mL) in amniotic fluid from Wistar rats orally exposed to mix-5 at three different doses.

Compound	Dose 5 mg/kg (n = 12)	Dose 15 mg/kg (n = 10)	Dose 50 mg/kg (n = 12)
Cypermethrin	2.1 (±1.2)	4.6 (±2.4)	20.3 (±14.6)
3-PBA (*)	20.5 (±8.3)	61.8 (±20.5)	213.3 (±54.8)
Bitertanol	19.9 (±24.3)	39.0 (±23.0)	158.4 (±92.8)
Propiconazole	0.3 (±0.3)	0.3 (±0.3)	2.1 (±2.5)
Terbuthylazine	2.1 (±1.4)	9.6 (±8.4)	39.6 (±36.1)
Desethylterbuthylazine (**)	22.8 (±8.7)	56.3 (±15.5)	142.6 (±36.2)
2-Hydroxyterbuthylazine (**)	0.1 (±0.03)	0.2 (±0.05)	0.5 (±0.1)
Malathion dicarboxylic acid (***)	2.7 (±2.6)	7.7 (±10.5)	23.6 (±23.4)

(*) metabolite of cypermethrin; (**) metabolite of terbuthylazine; (***) metabolite of malathion.

**Table 4:** Mean (±standard deviation) concentrations of pesticides and metabolites (ng/mL) in maternal urine from Wistar rats orally exposed to mix-3 at 3 different doses.

Compound	Dose 3 mg/kg (n = 6)	Dose 9 mg/kg (n = 6)	Dose 30 mg/kg (n = 6)
3-PBA (*)	619.0 (±160.1)	1,825.7 (±1,154.9)	3,753.3 (±811.6)
Bitertanol	1.3 (±0.5)	2.8 (±1.9)	10.4 (±2.6)
Propiconazole	0.10 (±0.11)	0.35 (±0.31)	2.2 (±0.9)

(*) metabolite of cypermethrin.

**Table 5:** Mean (±standard deviation) concentrations of pesticides and metabolites (ng/mL) in maternal urine from Wistar rats orally exposed to mix-5 at 3 different doses.

Compound	Dose 5 mg/kg (n = 4)	Dose 15 mg/kg (n = 6)	Dose 50 mg/kg (n = 4)
3-PBA (*)	463.0 (±145.8)	1,581.7 (±367.6)	3,880.0 (±1,498.7)
Bitertanol	1.3 (±0.60)	2.1 (±1.5)	16.7 (±24.8)
Propiconazole	0.36 (±0.10)	1.1 (±1.8)	12.9 (±20.3)
Terbuthylazine	0.65 (±0.16)	3.7 (±2.1)	22.4 (±20.3)
Desethylterbuthylazine (**)	138.3 (±52.8)	95.4 (±22.8)	69.9 (±61.0)
2-Hydroxyterbuthylazine (**)	60.7 (±23.6)	83.9 (±16.8)	68.4 (±64.5)
Malathion dicarboxylic acid (***)	215.8 (±16.8)	974.0 (±189.0)	1,488.5 (±440.2)

(*) metabolite of cypermethrin; (**) metabolite of terbuthylazine; (***) metabolite of malathion.

Cypermethrin was found at detectable concentrations in amniotic fluid (4.9–33.3 ng/mL). The ratios between cypermethrin and its metabolite 3-PBA were 0.12–0.18 and 0.07–0.10 for mix 3 and mix 5, respectively. Cypermethrin is known to be rapidly metabolized and mainly excreted in the urine as polar metabolites [20]. This was confirmed in our study by the high 3-PBA levels detected in maternal urine (range: 463–3,880 ng/mL). The fact that cypermethrin was found at detectable concentrations in amniotic fluid (4.9–33.3 ng/mL) is somehow surprising. Because of its lipophilic character, cypermethrin can be retained by adipose tissues, as observed in exposed rats [21]. A possible explanation is that the compound can be released to the maternal blood circulation and enter the fetal environment, as observed for a wide range of non-polar compounds [6,11,12]. Pyrethroid pesticides (*cis*- and *trans*-permethrin) have been detected at very low levels (<1 pg/mL) in cord serum and maternal serum in pregnant women and newborns, suggesting that some unmetabolized pyrethroids can also reach the fetal compartment [13]. 

The concentrations of terbuthylazine and its dealkylated metabolite desethylterbuthylzine were at comparable levels in the amniotic fluid and the urine, while much higher concentrations of its metabolite 2-hydroxyterbuthylazine were measured in urine. Terbuthylazine and other *s*-triazine pesticides such as atrazine, ametryne and terbutryne are metabolized to the N-monodealkylated and alkylhydroxylated compounds in *in vitro* metabolism studies [25]. There are no studies directed towards biological monitoring or investigation of *in vivo* metabolic paths of terbuthylazine. Since terbuthylazine follows the same biotransformation route as the other *s*-triazine compounds, the biomonitoring and *in vivo* metabolic data for atrazine may also apply for terbuthylazine [26]. Metabolic studies in rat and biological monitoring of atrazine exposure have shown that less than 2% of the parent compound is present in plasma and urine [17,27]. The results from urine samples in the present study show that terbuthylazine is extensively metabolized, since less than 2% of the parent compound is detected. The fraction of terbuthylazine detected in amniotic fluid is slightly higher than urine, ranging from 8% to 21% (from low to high dose).

Also the metabolite malathion dicarboxylic acid was present at much higher concentration in urine than amniotic fluid, which suggests that the unchanged compound might have been transported to the fetus.

**Figure 1:**
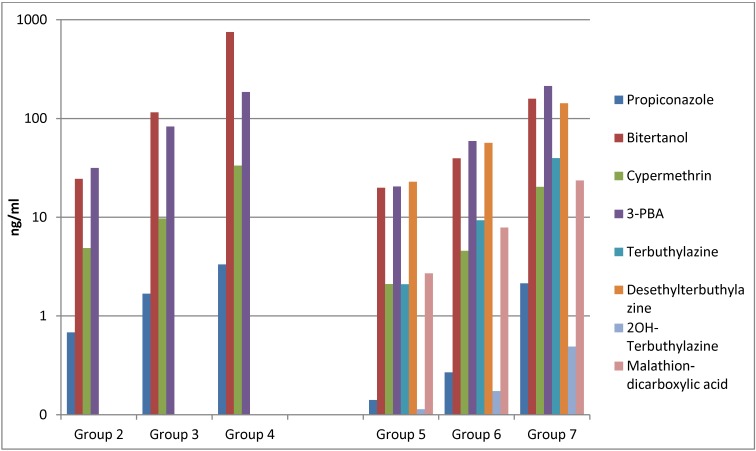
Average concentrations (n = 5) of the pesticides and their metabolites in amniotic fluid from Wistar rats. The y-axis is in logarithmic scale.

**Figure 2:**
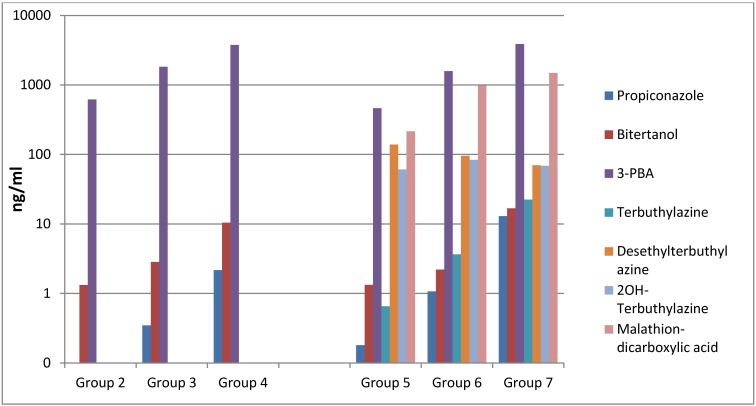
Average concentrations (n = 5) of the pesticides and their metabolites in maternal urine from Wistar rats. The y-axis is in logarithmic scale.

For all compounds a dose-dependent increase in amniotic fluid concentrations was consistent for both pesticide mixtures (Figure 1). The same trend was observed for the compounds detected in maternal urine with the exception of the metabolites desethylterbuthylazine, OH-terbuthylazine and malathion dicarboxylic acid (Figure 2).

## 4. Conclusions

The pesticides and metabolites investigated in the present study were found in amniotic fluid and urine—generally with increasing concentrations in correlation with increasing dosage of the rats. The pesticides detected in urine demonstrated the exposure as well as the ability of the rat to excrete these compounds. Moreover, the measurement of the substances in amniotic fluid indicated that the fetus was exposed to the pesticides as well as their metabolites. 

Upon exposure to mix 5 (bitertanol, propiconazole, cypermethrin, terbuthylazine, malathion), we found that the concentrations of bitertanol, propiconazole and cypermethrin in the amniotic fluid were relatively lower than if a mixture of the three pesticides (mix-3) were administered to the animals. This is a clear indication of ADME (absorption, distribution, metabolism and excretion) interactions *in vivo* between the five pesticides, which might lead to non-additive effects of the compounds.
